# Circulating Monocyte Chemoattractant Protein-1 in Patients with Cardiogenic Shock Complicating Acute Myocardial Infarction Treated with Mild Hypothermia: A Biomarker Substudy of SHOCK-COOL Trial

**DOI:** 10.3390/jcdd9080280

**Published:** 2022-08-20

**Authors:** Wenke Cheng, Georg Fuernau, Steffen Desch, Anne Freund, Hans-Josef Feistritzer, Janine Pöss, Petra Buettner, Holger Thiele

**Affiliations:** 1 Department of Internal Medicine/Cardiology, Heart Center Leipzig at University of Leipzig, 04289 Leipzig, Germany; 2 Medical Faculty, University of Leipzig, 04103 Leipzig, Germany; 3 Clinic for Internal Medicine II (Cardiology, Angiology, Diabetology, Intensive Care Medicine), Dessau Community General Hospital, 06847 Dessau-Rosslau, Germany

**Keywords:** monocyte chemoattractant protein-1, cardiogenic shock, acute myocardial infarction, mild therapeutic hypothermia

## Abstract

Background: There is evidence that monocyte chemoattractant protein-1 (MCP-1) levels reflect the intensity of the inflammatory response in patients with cardiogenic shock (CS) complicating acute myocardial infarction (AMI) and have a predictive value for clinical outcomes. However, little is known about the effect of mild therapeutic hypothermia (MTH) on the inflammatory response in patients with CS complicating AMI. Therefore, we conducted a biomarker study to investigate the effect of MTH on MCP-1 levels in patients with CS complicating AMI. Methods: In the randomized mild hypothermia in cardiogenic shock (SHOCK-COOL) trial, 40 patients with CS complicating AMI were enrolled and assigned to MTH (33 °C) for 24 h or normothermia at a 1:1 ratio. Blood samples were collected at predefined time points at the day of admission/day 1, day 2 and day 3. Differences in MCP-1 levels between and within the MTH and normothermia groups were assessed. Additionally, the association of MCP-1 levels with the risk of all-cause mortality at 30 days was analyzed. Missing data were accounted for by multiple imputation as sensitivity analyses. Results: There were differences in MCP-1 levels over time between patients in MTH and normothermia groups (*P* for interaction = 0.013). MCP-1 levels on day 3 were higher than on day 1 in the MTH group (day 1 vs day 3: 21.2 [interquartile range, 0.25–79.9] vs. 125.7 [interquartile range, 87.3–165.4] pg/mL; *p* = 0.006) and higher than in the normothermia group at day 3 (MTH 125.7 [interquartile range, 87.3–165.4] vs. normothermia 12.3 [interquartile range, 0–63.9] pg/mL; *p =* 0.011). Irrespective of therapy, patients with higher levels of MCP-1 at hospitalization tended to have a decreased risk of all-cause mortality at 30 days (HR, 2.61; 95% CI 0.997–6.83; *p* = 0.051). Conclusions: The cooling phase of MTH had no significant effect on MCP-1 levels in patients with CS complicating AMI compared to normothermic control, whereas MCP-1 levels significantly increased after rewarming. Trial registration: NCT01890317.

## 1. Introduction

Cardiogenic shock (CS) is the most severe complication in hospitalized patients with acute myocardial infarction (AMI) [1]. Immediate revascularization of the infarct-related coronary artery is still the only treatment proven effective in a randomized clinical trial [2]. Despite the widespread implementation of early revascularization, mortality in AMI patients complicated by CS remains at 50% [3]. Early reperfusion not only salvages ischemic cardiomyocytes but is also often accompanied by reversible and irreversible damage to cardiomyocytes, known as reperfusion injury. During this process, inflammation and inflammatory cell infiltration, together with activation of intrinsic and adaptive immune responses, are the hallmarks of AMI and reperfusion injury [4,5]. Moreover, CS is a systemic syndrome, and new therapeutic strategies with global systemic effects are needed for treatment and prognosis of CS after AMI. Mild therapeutic hypothermia (MTH) exhibited no hemodynamic benefits in terms of cardiac power index (CPI) in patients with CS complicating AMI in the randomized SHOCK-COOL trial [6]. However, little is known about the impact of MTH on inflammation in patients with CS complicating AMI. MTH as a treatment option in AMI can reduce myocardial reperfusion injury and down-regulate the inflammatory response, as demonstrated in vitro and in animal models [7,8,9,10]. There, MTH improved metabolic parameters in CS and reduced the release of pro-inflammatory factors [11]. Therefore, we conjectured that the effects of MTH on inflammation may similarly be present in humans.

Monocyte chemoattractant protein-1 (MCP-1) is a chemotactic protein that mediates monocyte recruitment to sites of inflammation and injured tissues, regulates monocyte and lymphocyte phenotypes and promotes fibrous tissue deposition and angiogenesis [12]. Moreover, during the inflammatory phase of infarct healing, MCP-1 is markedly induced in the infarcted myocardium and mediates the recruitment of macrophages to the infarcted area for the clearance of necrotic cardiomyocytes, thereby playing an important role in infarct healing and post-infarct remodeling [13]. MCP-1 also exerts pro-apoptotic effects on cardiomyocytes [14]. Two previous sub-analyses based on multicenter trials in acute coronary syndromes revealed that MCP-1 can be identified as an independent risk stratification biomarker in addition to C-reactive protein (CRP) and brain natriuretic peptide (BNP) [15,16]. Meanwhile, there is clinical evidence that MCP-1, which reflects the inflammatory response in patients with AMI complicated by CS, is strongly expressed in the early phase (0–4 h), and has prognostic value in assessing the clinical outcomes [17,18]. Consequently, MCP-1 may provide a new therapeutic or prognostic perspective for myocardial infarction and ischemic cardiomyopathy [13,19]. However, no previous studies have investigated the effect of MTH on MCP-1 in patients with CS complicating AMI. Therefore, we conducted a biomarker study to analyze the effect of MTH on MCP-1 levels.

## 2. Methods

### 2.1. Patients and Study Design

This is a sub-study of the SHOCK-COOL Trial (ClinicalTrials.gov (accessed on 16 August 2022) Identifier: NCT 01890317), where hemodynamic effects of MTH on CPI were analyzed in CS complicating AMI. The design and main results of the SHOCK-COOL trial have been described previously [6]. Briefly, 40 patients with CS complicating AMI were enrolled at the Heart Center Leipzig from July 2012 to March 2015 and assigned to MTH and normothermia at a 1:1 ratio. The target temperature in the MTH group was 33 °C. Cooling was initiated in the cardiac catheterization laboratory and maintained for 24 h after reaching the target temperature. Thereafter, patients were rewarmed to 37 °C at a rate of 0.25 °C/h. All patients underwent mechanical ventilation. Exclusion criteria were as follows, (1) patients aged over 90 years; (2) out of hospital resuscitation with indication for MTH; (3) mechanical complications after AMI; and (4) those with CS lasting for more than 12 h. Blood sample collection and cardiac catheterization were performed immediately after admission. The study was approved by the local Ethical Committee (Medical Faculty, University Leipzig, registration number 230-12-21052012) and conducted in accordance with the Declaration of Helsinki. The written informed consent process has been described in detail previously [6].

### 2.2. Laboratory Measurements

Blood samples were collected at predefined time points at the day of admission/day 1, day 2 and day 3. Serum and plasma were obtained by centrifugation at 1000 g for 10 min, and aliquots were stored at −80 °C. Blood biochemical parameters including creatine kinase (CK), creatine kinase-MB, creatinine, white blood cell count and C-reactive protein were measured by standardized laboratory procedures. MCP-1 was determined using a commercial enzyme-linked immunosorbent assay (ELISA) kit (Catalog number: DY279, R&D systems, Abingdon, UK) according to the manufacturer’s instructions. Samples were assayed in duplicate. Quality control was performed on the ELISA results, and samples with coefficient of variation (CV) values > 25% were excluded from further analysis. Samples with values below standard range were set to be zero for statistical analysis.

### 2.3. Statistical Analysis

Categorical data are expressed as counts and proportions. Since most variables were not normally distributed, all continuous variables are expressed as median with interquartile range (IQR). Comparisons of two groups of dichotomous variables were analyzed by Fisher’s exact test, whereas continuous variables were compared using the Mann–Whitney test. Spearman’s rank correlation was employed to investigate the correlation between MCP-1 levels and different clinical characteristics and biomarkers. The primary outcomes were the differences in MCP-1 levels over time between and within the MTH and normothermia groups. For the differences in MCP-1 levels between the MTH and normothermia groups over time, the mixed linear model (MLM) was adopted, and data were fitted by a mixed model (treatment modality group as a factor) with random intercepts. In this model, baseline values were adjusted, and time was taken as a continuous variable, while the *p*-value was calculated for the interaction between the two treatment groups. Moreover, in the MLM, the data were analyzed using maximum likelihood induction suitable for dealing with designs featuring substantial dropout rates [20,21]. Apart from that, differences in MCP-1 levels between the two groups were expressed as median difference and 95% confidence interval (CI), calculated by the Hodges–Lehmann method. For the differences in MCP-1 levels for the three days (day 1, 2 and 3) within the MTH or normothermia groups, nonparametric Kruskal–Wallis one-way analysis of variance (ANOVA) on ranks with Dunn’s multiple comparison tests were adopted. Additionally, to maximize statistical power and minimize possible bias due to exclusion of missing data, a sensitivity analysis was conducted to account for missing MCP-1 data on day 1, 2, and 3 by multiple imputation, which was carried out based on the predictive mean matching (PMM) algorithm by 5 replications and the Markov chain Monte Carlo (MCMC) method [22]. All-cause mortality after 30 days was regarded as a secondary outcome. Samples were divided into two groups irrespective of treatment arm, according to the median MCP-1 level over three days, and time-to-death was analyzed by the Kaplan–Meier method with log-rank test. Statistical analyses were performed using GraphPad prism (version 9.0; GraphPad Software, San Diego, CA, USA) and SPSS (version 26; SPSS Inc., Chicago, IL, USA). All tests were two-tailed, and *p* < 0.05 was considered statistically significant.

## 3. Results

**Figure 1:**
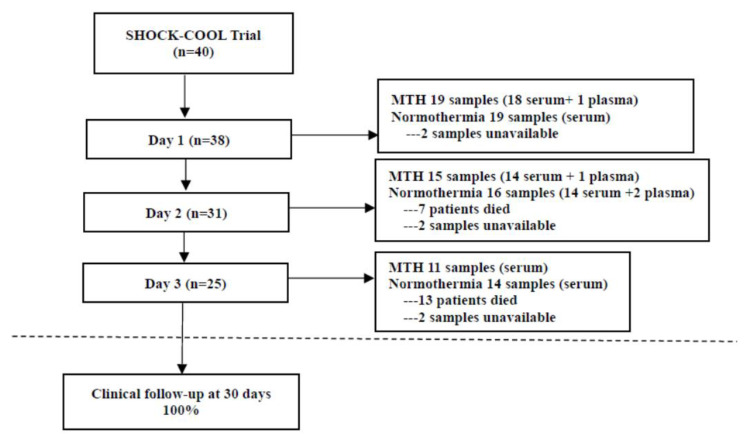
Study flow. SHOCK-COOL, the randomized trial of mild hypothermia for cardiogenic shock. MTH, mild therapeutic hypothermia.

In the SHOCK-COOL trial, 40 patients aged 50–87 (median 76) years with CS complicating AMI were randomized to MTH or normothermia. Overall, 38 patients (MTH 19 and normothermia 19) had blood samples available for testing of MCP-1 levels at baseline/day 1. A total of 31 samples (MTH 15 and normothermia 16) were accessible at day 2 and 25 (MTH 11 and normothermia 14) on day 3 (Figure 1). Ultimately, following quality control, 35 samples from day 1, 31 samples from day 2 and 23 samples from day 3 were statistically analyzed in the current study. As shown in Table 1, there were no significant differences in demographic and clinical characteristics between the MTH and normothermia groups on day 1, day 2 and day 3, except that CK in the normothermia group was higher than in the MTH group on day 2.

### MCP-1 Levels in MTH and Normothermia Groups

**Figure 2:**
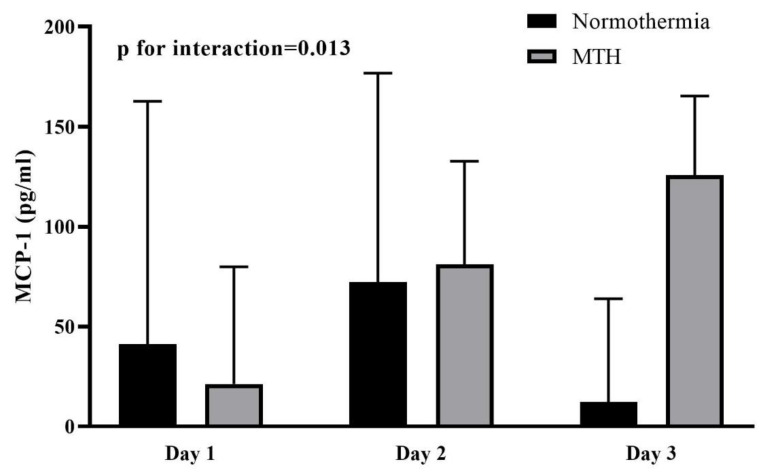
MCP-1 levels of patients in cardiogenic shock after acute myocardial infarction treated by either mild therapeutic hypothermia (MTH) or normothermia 1, 2 and 3 days following hospitalization. Box and whisker plots show median and interquartile range.

During the three days after admission, MCP-1 levels gradually increased in the MTH group, whereas in the normothermia group levels first increased (day 1 to day 2) and then decreased (day 2 to day 3) (Figure 2). There were differences in MCP-1 levels over time between the MTH and normothermia groups (*P* for interaction = 0.013; Figure 2). No significant differences in MCP-1 levels between MTH and normothermia groups on day 1 (MTH 21.2 [IQR 0.25–79.9] vs. normothermia 41.2 [IQR 9.2–162.7] pg/mL; median difference, −15.17 pg/mL; 95% CI, 63.94 to 12.67; *p =* 0.337; Table 2 and Table 3) and day 2 (MTH 81.1 [IQR 18.9–132.7] vs. normothermia 72.3 [IQR 8.3–176.8] pg/mL; median difference, 8.80 pg/mL; 95% CI, −64.21 to 65.41; *p =* 0.984) were observed. However, MCP-1 levels in the MTH were higher than in normothermia on day 3 (MTH 125.7 [IQR 87.3–165.4] vs. normothermia 12.3 [IQR 0–63.9] pg/mL; median difference, 97.67 pg/mL; 95% CI, 28.16 to 150.2; *p =* 0.011). Spearman correlation analysis showed that MCP-1 levels on day 1 were not significantly correlated with gender (*p* = 0.65), age (*p* = 0.39), smoking (*p* = 0.99), DM (*p* = 0.59), BMI (*p* = 0.95), CK-MB (*p* = 0.37), CRP (*p* = 0.14), white blood cell (*p* = 0.82), creatinine (*p* = 0.52) or CK (*p* = 0.27), while the results were consistent with day 2 and day 3. Additionally, five new sets of data were obtained by multiple imputation of the missing data, and sensitivity analyses demonstrated that the results remained consistent before and after imputation (Table 2).

Multiple comparisons within groups showed that MCP-1 levels steadily increased in the MTH group and were highest on day 3 (day 1 vs day 3: 21.2 [IQR 0.25–79.9] vs. 125.7 [IQR 87.3–165.4] pg/mL; mean rank difference, −15.28; *p* = 0.006; Table 3). Nevertheless, there were no significant differences in MCP-1 levels between day 1, 2 and 3 in the normothermia group (*P* > 0.05; Table 3). These results were consistent before and after imputation.

**Figure 3:**
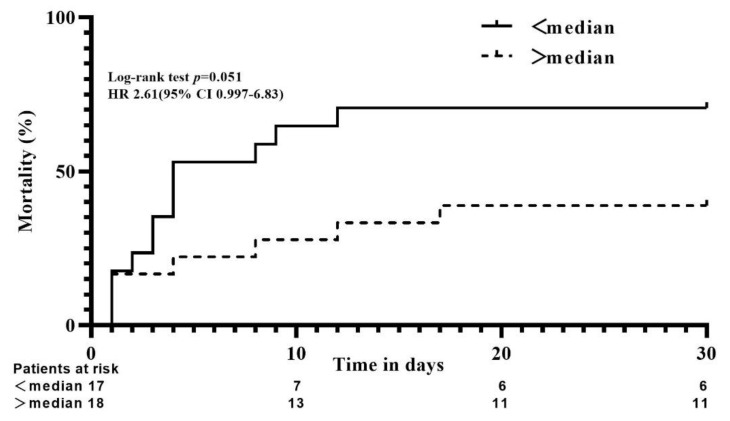
Kaplan–Meier analysis for time to death within the first 30 days in cardiogenic shock complicating acute myocardial infarction patients with MCP-1 levels < median (black solid line) and > median (black dashed line) on day 1.

As a secondary outcome, we exploratively assessed the association of MCP-1 levels and 30-day all-cause mortality. Irrespective of therapy, we analyzed all patients according to their median MCP-1 levels at baseline/day 1 and divided them into <median and >median groups. Patients with MCP-1 levels below the median on day 1 tended to have an increased risk of all-cause mortality at 30 days (HR, 2.61; 95% CI 0.997–6.83; *p* = 0.051; Figure 3).

## 4. Discussion

This study is the first to assess the effect of MTH on the plasma levels of MCP-1 in patients with CS complicating AMI. The main results suggest that the dynamics of change of MCP-1 levels are different between MTH and normothermia. In the first three days after admission, MCP-1 levels in the normothermia group first increased and then decreased, whereas MCP-1 levels in the MTH group increased continuously and were higher on day 3 than those in the normothermia group. Meanwhile, the dynamic changes in circulating MCP-1 levels were relatively independent and were not explicitly correlated with patient clinical characteristics or other biomarkers.

Currently, the effect of MTH on inflammation has been mostly studied in patients after cardiac arrest, while studies in patients with CS complicating AMI are still limited. In the present analysis of the SHOCK-COOL trial, the MTH group had a cooling phase on the first day, a rewarming phase on the second day and a post-rewarming phase on the third day. Body temperature in the MTH group was significantly lower than that of the normothermic control group for the first 40 h and comparable after 48 h. MCP-1 levels in both groups were comparable in the first 2 days, which is consistent with the previous results of Beurskens et al., who studied patients with out-of-hospital cardiac arrest [23]. In addition, MCP-1 levels increased in MTH until day 3, where they were also higher than in the normothermia group. The underlying mechanisms remain unclear but may be related to the pro-inflammatory response after rewarming. We speculate that MCP-1 may be “temperature-sensitive”, as there was a trend of increasing MCP-1 levels in the MTH group in line with temperature changes, differing from the normothermia group. Indeed, several studies have suggested that rewarming alters inflammatory homeostasis, activating the complement system and promoting inflammatory responses, depending on the rate and duration of rewarming [24,25,26,27]. Meanwhile, one study has also shown that prolonged hypothermia attenuates the inflammatory response in patients after rewarming, which may be associated with a decrease in leukocyte count and function [28].

Over the past decade, many studies have intensively investigated the suitability of biomarkers in the prognosis of outcomes in CS patients [29,30,31,32], whereas MCP-1 has not been studied yet. In the present analysis, a trend towards reduced 30-day all-cause mortality in patients with higher MCP-1 levels on day 1 was observed regardless of treatment modality. This is inconsistent with the results of several previous large clinical studies [15,33,34]. These discrepancies might be explained by the following. First, the small sample size is not powered to detect differences in mortality and, therefore, the results of our exploratory analysis may be just a play of chance. Second, this discrepancy may be due to the differences in the timing of blood collection. MCP-1 might play different roles at different stages after AMI and evaluating one chemokine as “good” or “bad” from one stage alone does not provide a general overview of its function. Moreover, it has been reported that there may be a “time window” in the orchestration of MCP-1 in the myocardial stress response, delimiting tissue injury and promoting tissue repair by providing cytoprotective signals in the early stage and further exacerbating adverse remodeling of injured myocardial tissue by providing delayed signals in the later stage [35]. Yet, our small study is not able to answer these questions regarding prognosis. It is well known that ischemia-reperfusion injury after AMI triggers a complex inflammatory response with cytokine release and inflammatory leukocyte infiltration of the infarcted area [36]. MCP-1 is rapidly upregulated in the infarcted myocardium and is mainly expressed by endothelial cells and infiltrating leukocytes [37]. Studies have demonstrated that short-term expression of pro-inflammatory factors is involved in cardiac repair by activating leukocyte recruitment to the infarct zone and subsequent clearing of necrotic cells and matrix debris [13,38]. Furthermore, in animal models a potential protective role for acute MCP-1 overexpression in the heart was proposed, as early MCP-1 overexpression was shown to reduce infarct size [39]. MCP-1 overexpression further improved left ventricular pressure [40] and protected hypoxic cardiomyocytes from death [41,42]. However, under prolonged chronic inflammatory conditions, chronically elevated MCP-1 levels can lead to myocardial hypertrophy and dilatation, left ventricular hypoperfusion and atherosclerosis [43,44]. Summarizing, this sub-study provides a new perspective on the treatment of patients with CS complicating AMI, and larger studies are needed to further evaluate the application value of different stages of MCP-1 in this population.

## 5. Limitations

This analysis has several limitations. 1) The study population was Caucasian and more than 70% of the patients were overweight and older than 70 years, and the role of adipose tissue in changes in MCP-1 was unclear. Therefore, results might not be transferable to other populations. Additionally, the extent of coronary lesions was similar in both groups and the utilization rate of various assist devices (e.g., extracorporeal live support and intra-aortic balloon counter pulsation) was very low (<10%), thus the effect of these factors on MCP-1 was limited. 2) Due to the limited sample size, the negative results in this study might be susceptible to low power. Meanwhile, the proportion of missing data was higher on day 2 and 3 (22.5% and 42.5%), and although consistent results were obtained by both complete case analysis and multiple imputation to maximize the validity of our statistical methods, bias due to missing data cannot be excluded. 3) In line with the original study, the present study was not powered to investigate mortality. Therefore, the presented short-term mortality rates are exploratory only.

## 6. Conclusions

To conclude, the cooling phase of MTH had no significant effect on MCP-1 in patients with CS complicating AMI compared to the normothermic control group, whereas MCP-1 levels were significantly increased after rewarming.

## Figures and Tables

**Table 1:** Demographic and clinical characteristics of patients during three days of hospitalization.

	Day 1			Day 2			Day 3		
	MTH (n = 18)	NTH (n = 17)	*p*-Value	MTH (n = 15)	NTH (n = 16)	*p*-Value	MTH (n = 10)	NTH (n = 13)	*p*-Value
Age, years (IQR)	77 (71–81)	75 (68–80)	0.58	76 (71–78)	75.5 (70.8–82)	0.61	76 (59–77)	74 (65–82)	0.64
Male, n (%)	10 (55.6)	14 (82.4)	0.15	10 (66.7)	11 (69)	>0.99	6 (60)	9 (69.2)	0.69
Active smoker, n (%)	4 (22.2)	4 (23.5)	> 0.99	4 (26.7)	3 (18.8)	>0.99	2 (20)	4 (30.7)	0.66
Diabetes mellitus, n (%)	7 (38.9)	3 (17.7)	0.26	5 (33.3)	3 (18.8)	0.43	3 (30)	3 (30.8)	>0.99
CK (U/L) (IQR)	5.8 (3–22.1)	19.8 (6.4–40)	0.12	5.8 (1.9–17.6)	19 (9.8–35.3)	0.027	3.8 (2.6–7.5)	16 (4.1–32)	0.058
BMI, kg/m^2^ (IQR)	27.6 (23.5–33.7)	27.8 (25–31)	0.98	27(23.6–31.1)	27.8 (25–30.7)	0.78	28 (23–32.6)	29 (25.3–31)	0.87
White blood cell, 10^9^ /L (IQR)	16 (12.9–19)	14 (9.2–17.8)	0.18	16 (13.4–18)	14 (10.5–17.8)	0.29	17.4 (14–20.6)	13 (8–17.6)	0.08
Creatinine, µmol/L (IQR)	123 (75–227)	154 (95–259)	0.33	128 (70–197)	147 (95–223)	0.36	114 (63–146)	144 (91–228)	0.21
CRP (IQR)	30.7 (3.5–78)	10.4 (6.8–63)	0.81	7.6 (2.7–82)	14.3 (7.2–64)	0.48	9.8 (2–102)	22 (6.7–63.7)	0.65
Maximum CK-MB, U/L(IQR)	2.6 (1.4–4.9)	3.9 (1.8–9.2)	0.37	2.7 (1.4–8.3)	2.6 (1.8–9)	0.78	2.3 (1.2–6.2)	2.8 (1.4–6.9)	0.69

MTH, mild therapeutic hypothermia. NTH, normothermia. IQR, interquartile range. CK, creatine kinase. CK-MB, creatine kinase-MB. CRP, C-reactive protein.

**Table 2:** Differences in MCP-1 between the MTH and normothermia groups on day 1, day 2 and day 3.

	Day 1		Day 2		Day 3		
MCP-1 (pg/mL)	MTH vs. NTH *	*p*-Value	MTH vs. NTH *	*p*-Value	MTH vs. NTH *	*p*-Value	*P* for Interaction ^&^
Before imputation	−15.17 (-63.94 to 12.67)	0.337	8.80 (−64.21 to 65.41)	0.984	97.67 (28.16 to 150.2)	0.011	0.013
After imputation 1	−15.17 (−63.5 to 9.64)	0.271	12.05 (−51.74 to70.55)	0.753	114.2 (68.53 to 150.6)	<0.001	<0.001
After imputation 2	−18.46 (−62.88 to 10.18)	0.290	17.09 (−32.71 to 65.43)	0.473	99.11 (61.46 to150.6)	<0.001	0.001
After imputation 3	−6.24 (−35.34 to 28.87)	0.643	14.99 (−32.71 to70.55)	0.515	90.79 (47.01 to 139.4)	<0.001	<0.001
After imputation 4	−2.54 (−40.89 to 13.40)	0.721	0 (−64.21 to 70.55)	0.899	93.19 (28.51 to 147.1)	<0.001	0.001
After imputation 5	−7.03 (−35.34 to 22.99)	0.515	4.55 (−63.13 to 70.55)	0.712	97.84 (55.5 to 146.4)	<0.001	0.003

* Results are expressed as median difference and 95% confidence interval. ^&^ Interaction of MCP-1 in the MTH and normothermia groups over time. MTH, Mild therapeutic hypothermia. NTH, normothermia. MCP-1, monocyte chemoattractant protein-1.

**Table 3:** Multiple comparisons of MCP-1 differences within the MTH and normothermia groups.

	MTH Group						NTH Group					
MCP-1 (pg/mL)	Day 1 Median (IQR)	Day 2 Median (IQR)	Day3 Median (IQR)	Dunn’s Test *			Day 1 Median (IQR)	Day 2 Median (IQR)	Day3 Median (IQR)	Dunn’s Test *		
				Day 1 vs 2	Day 1 vs 3	Day 2 vs 3				Day 1 vs 2	Day 1 vs 3	Day 2 vs 3
Before multiple Imputation	21.2 (0.25–79.9)	81.1 (18.9–132.7)	125.7 (87.3–165.4)	–7.81; *p* = 0.224	–15.28; *p* = 0.006	−7.45; *p* = 0.433	41.2 (9.2–162.7)	72.3 (8.3–176.8)	12.3 (0–63.9)	−2.45; *p* > 0.99	5.62; *p* = 0.762	8.07; *p* = 0.318
After multiple imputation 1	21.2 (0.08–85.8)	88.2 (62–151.6)	155.2 (98.2–159.4)	−11.30; *p* = 0.121	−21.93; *p* < 0.001	−10.63; *p* = 0.162	52.4 (6.2–115.8)	72.3 (21.6–194.6)	10.4 (0.1–37.7)	−3.53; *p* > 0.99	9.0; *p* = 0.307	12.53; *p* = 0.07
After multiple imputation 2	21.2 (0.08–85.8)	88.2 (59.2–132.7)	158.7 (99.3–188.3)	−11.10; *p* = 0.132	−21.53; *p* < 0.001	−10.43; *p* = 0.176	52.4 (16.6–115.8)	72.3 (17.9–123.1)	35.6 (0.4–77.1)	−2.70; *p* > 0.99	6.60; *p* = 0.693	9.30; *p* = 0.275
After multiple imputation 3	37.1 (0.4–95.1)	81.1 (28.9–130.2)	125.7 (97.8–183.4)	−8.80; *p* = 0.332	−19.10; *p* = 0.002	−10.30; *p* = 0.186	35.3 (16.6–110.6)	58.8 (5.1–123.1)	23.0 (0.1–77.1)	−1.20; *p* > 0.99	5.85; *p* = 0.864	7.05; *p* = 0.601
After multiple imputation 4	21.2 (0.3–76.9)	106.9 (28.9–151.6)	155.2 (66.1–177.4)	−13.15; *p* = 0.0513	−20.30; < 0.001	−7.15; *p* = 0.584	28.6 (0.8–110.6)	89.8 (8.3–194.6)	10.4 (0.4–77.1)	−7.15; *p* = 0.581	2.43; *p* > 0.99	9.58; *p* = 0.245
After multiple imputation 5	29.1 (0.4–85.8)	88.2 (28.9–151.6)	125.7 (97.8–159.4)	−11.05; *p* = 0.135	−18.50; *p* = 0.002	−7.45; *p* = 0.53	35.3 (15.2–110.6)	72.3 (5.1–237.5)	12.3 (0–37.6)	−2.38; > 0.99	9.05; *p* = 0.297	11.43; *p* = 0.112

MTH, Mild therapeutic hypothermia. NTH, normothermia. MCP-1, monocyte chemoattractant protein-1. IQR, interquartile range. * Dunn’s multiple comparison test expressed by mean rank difference and *p*-value.
